# Orthodontic Treatment of Palatally Impacted Maxillary Canines with the Use of a Digitally Designed and 3D-Printed Metal Device

**DOI:** 10.3390/dj11040102

**Published:** 2023-04-12

**Authors:** Georgios Vasoglou, Ioannis Lyros, Athanasia Patatou, Michail Vasoglou

**Affiliations:** 1 Private Orthodontic Practice, 17676 Athens, Greece; 2 Department of Orthodontics, School of Dentistry, National and Kapodistrian University of Athens, 11527 Athens, Greece

**Keywords:** impacted canines, cone-beam CT, intraoral scanner, 3D printing

## Abstract

The purpose of this article is to present a computer designed and 3D-printed metal device, which was used for the surgical exposure and orthodontic treatment of maxillary palatally impacted canines. In two cases which presented a palatally impacted canine, a Cone-Beam Computed Tomography (CBCT) was acquired and an intraoral scanning was performed, to determine the exact location of the canine. Based on a digital model, a device leaning on the teeth and mucosa was designed to serve as a guiding tool for the oral surgeon to expose the crown of the canine and help the orthodontist to provide proper traction. The device was then 3D-printed in biocompatible dental alloy and placed in the patients’ mouth. After the surgical exposure of the canine’s crown in both cases, a gold chain apparatus was bonded on and it was mounted on the metal projection of the device through an elastic chain. Within 3 months of traction, the crown of the canines appeared in the patients’ palate to the exact location that was predicted and guided. A 3D-designed and manufactured metal device, with information acquired by CBCT and intraoral scanning, can be used for the exposure and traction of palatally impacted canines.

## 1. Introduction

Unerupted maxillary canines appear in approximately 0.8%–2% of the general population [1] and 85% of them are palatally impacted [2]. The causes of impaction of the maxillary permanent canines are local pathology, local obstruction, or it is genetically determined and linked to anomalous or missing lateral incisors [3]. Some studies claim that the latter cause is controversial [4] and others do not support it and conclude that environmental or epigenetic variables affect the phenotype [5]. Furthermore, certain skeletal abnormalities such as Sella turcica bridging (SB), ponticulus posticus (PP), atlas posterior arch deficiency (APAD) and an elongated pterygopalatine fissure have been found to be significantly associated with maxillary canine impaction [6,7]. Impacted canines are a challenging pathologic modality and this is the reason for establishing certain treatment protocols in order to treat them safely and successfully.

Two possible strategies in uncovering these impacted teeth have been advocated: the open-eruption technique and the closed one [8]. In the first technique, the mucosa and bone overlying the impacted canine are removed and a surgical pack is placed, while in the second, a mucoperiosteal flap is reflected, the impacted tooth is revealed after bone removal and an attachment with a chain is bonded on the crown’s surface. After that, the flap is sutured back and the chain is mounted properly on the fixed appliances. The latter technique has been suggested to lead to better periodontal status of the impacted tooth, when it is restored to its proper position [9]. However, some studies claim that there is no difference either in the surgical outcome between the two techniques [10], or the periodontal outcomes and aesthetic appearance [11], while others concluded that open surgical exposure seems to be superior in terms of treatment duration and ankylosis risk, over the closed technique [12].

Two-dimensional radiographic images such as panoramic, periapical and bite-wing radiographs, have been used for many years in detecting and evaluating the impacted canines. A classification system, utilizing panoramic radiographs, which includes the determination of the sector of the upper canine’s crown position in relation to the adjacent teeth, the distance of the canine’s crown from the occlusal plane and the canine’s inclination to the upper midline, was introduced by Ericson and Kurol [2]. The importance of the cephalometric records in diagnostic procedures was also highlighted by Quinzi et al. [13]. However, new technology, such as cone-beam computed tomography (CBCT), can help in precisely locating the impacted canines in three dimensions [14,15]. In addition, 2D images have a disadvantage compared to CBCT as to the detection of root resorption of the adjacent incisor [16,17], or the possible dilaceration of the canine root [18].

The exact location of the impacted canines in the 3D images (i.e., sagittal, coronal and axial) and their contact with other teeth, allows clinicians to determine the direction of traction, to avoid injury of the adjacent teeth, as well as to ensure better surgical access [19]. Consideration should be given to the amount of ionizing radiation, as to the risk of deleterious effects. The dose received by the patient is related to the field of view (FOV), the scan time and the number of projections, while the mAs (Milliampere-seconds) have an additional role in reducing the dose [20].

Several appliances have been introduced for the traction of impacted canines, designed in order to improve the force delivery on them. The “ballista spring” system, for impacted teeth (a 0.014-, 0.016-, or 0.018-inch round wire, which accumulates its energy by being twisted on its long axis), was presented by Jacoby [21].

An extrusion spring for palatally impacted canines, using a prefabricated 0.018-inch stainless steel arch-wire, was devised by Oppenhuizen [22]. The directional force springs, called “Kilroy I and Kilroy II” for palatally and buccally impacted canines, respectively, were introduced by Bowman and Carano [23]. Traction, using the elastic force from the eyelets bonded to the crown of the impacted canine, was applied by Sivakumar et al. [24]. Microscrews and elastic force for guiding the impacted tooth to its place were used by Haydar et al. [25]. An auxiliary strut, soldered to a lingual arch with a coiled eyelet forming a fairlead at its end, was designed by Johnson [26], for applying pressure at a biologically efficient level to an impacted tooth, with mechanics adjustable to all three planes of space. The use of magnets, to deliver effective force in order to treat impacted canines, was proposed by Vardimon et al. [27]. A modified transpalatal arch (TPA), which incorporated a stretched arm at the traction side, was used by Nakandakari et al. [28], for treating palatally impacted canines.

The recommended optimum force level for extrusion is 0.3 to 0.4 N [29], while continuous and constant forces are important for the maximum biological response and minimal tissue damage. Consequently, using an appliance with knowledge of the force system is an important step for evidence-based treatment, which is essential for achieving better results [30,31].

A method to deliver physiologic force for the extrusion of palatally impacted maxillary canines was presented by Tepedino et al. [32], using a stainless steel cantilever welded to a transpalatal bar, where the free end of the cantilever wire was rolled multiple times. Finally, a new appliance to treat impacted maxillary incisors, incorporating a Nance arch, a traction hook, an M bracket and molar bands with buccal tubes, was introduced by Peng et al. [33], which can be modified for the traction of impacted canines.

At present, given that digital images and 3D printing have become available and easy to use, it is apparent that these technologies can facilitate the procedures involving impacted canines [34]. This article aims to introduce a metal device which is digitally designed with the help of CBCT and intraoral scanning and is 3D printed. This device, in contrast to complicated mechanics, can help in easily detecting the crown of palatally impacted canines during surgery. Furthermore, it accomplishes the desired direction of traction in the initial phase, with the aim of favorably repositioning the tooth in the palate, before giving traction to a buccal direction.

## 2. Materials and Methods

**Figure 1:**
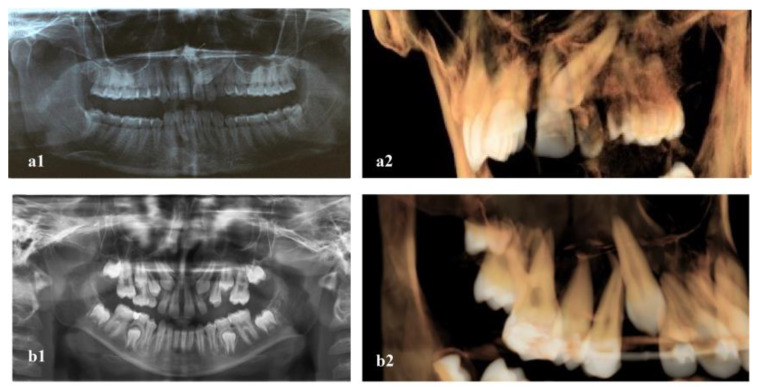
Panoramic and CBCT imaging of the two patients presenting palatally impacted canines ((**a1**,**a2**): patient 1, (**b1**,**b2**): patient 2).

A 23-year-old female patient (patient 1) and a 14-year-old male patient (patient 2), presented with a palatally impacted canine (tooth 23 and 13, respectively). Since the canines in both cases were in close proximity with the roots of the lateral incisors (sector 5 and 3, respectively, according to Ericson and Kurol [2]), there was a need for a precise direction of the orthodontic traction. It was decided that the canines would be initially guided in a more favorable position in the palate and then traction to a labial direction would be delivered on them. These were the criteria for treating the specific impacted canines with the help of a novel 3D-printed metal device, bonded on the patient’s teeth, besides the conventional fixed orthodontic appliances. A written consent, where the procedure was fully described, was provided by the patients (or their representatives). After diagnostic procedures, the fixed appliances were fitted on the upper arch in order to obtain the demanded space for the canines. Besides the conventional 2D radiograph (panoramic x-ray), a cone-beam computed tomography (CBCT) was acquired (Planmeca ProMax^®^ CBCT system, Planmeca Oy, Helsinki, Finland, 90 kVp/4–10 mA, 200–400 μm voxel size), in order to determine the exact location of the canines (Figure 1).

**Figure 2:**
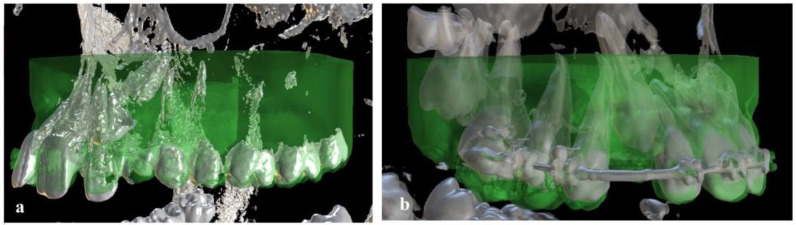
The digital models of the two cases produced by combining DICOM and .STL files (green area). ((**a**): patient 1, (**b**): patient 2).

An intraoral scanning of the upper jaw of the patients was also performed (CS 3600, digital oral scanner, Carestream Dental, LLC, Atlanta, GA 30339, USA). An open-eruption technique was chosen in agreement with the surgeon for both cases. DICOM and .STL files were uploaded on an open-source software (Blue Sky Plan^®^, Version 4.9.4, Blue Sky Bio, LLC, Libertyville, IL 60048, Illinois, USA). This is a computer software for viewing and reformatting images created by computerized tomography. The two files were combined with several matching points onto a digital model in which the roots, bone, mucosa, and teeth could be evaluated at the same time (Figure 2). 

**Figure 3:**
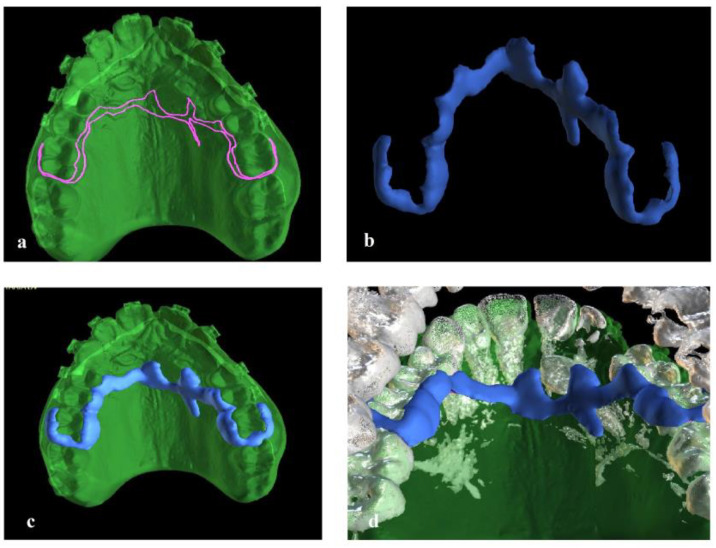
(**a**,**b**): The design (purple color) and the .STL file (blue color) of the device. (**c**,**d**): The device on the digital model (patient 1).

In order to spot the exact region of the palatal mucosa to be removed, a device leaning on the 1st molar’s (or 2nd premolar’s) buccal and palatal surfaces and palatal mucosa was designed on the digital model (Figure 3). The device design incorporated a semi-circular construction, which indicated the location of the crown of the impacted canine and the exact point on which the eyelet of the chain apparatus would be bonded. All this information was determined from the underlying CBCT image. In this way, it served as a guiding tool for the oral surgeon to remove the exact amount of overlying mucosa and bone and expose the crown of the canine. The device also incorporated a projection, on which an elastic chain was intended to be mounted, so as to provide the desired and most convenient direction of traction to the canine.

**Figure 4:**
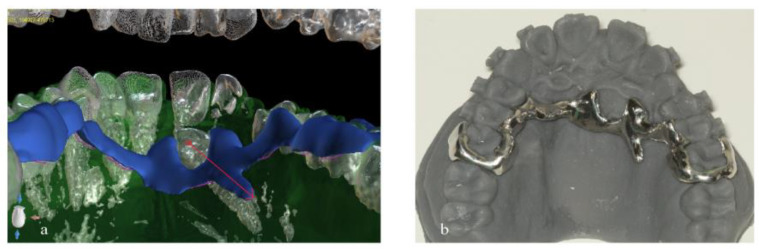
(**a**): The device (blue color), indicating the crown of the canine and the direction of traction (purple arrow). (**b**): The device 3D-printed in biocompatible dental alloy.

The device was then 3D-printed using Selective Laser Melting (SLM) technology, in biocompatible dental alloy (Co 61%, Cr 28,1%, M 5,3%, W 5%, Cobalt alloys-Audental Biomaterial Co, Ltd., Shenzhen, China), (Figure 4).

**Figure 5:**
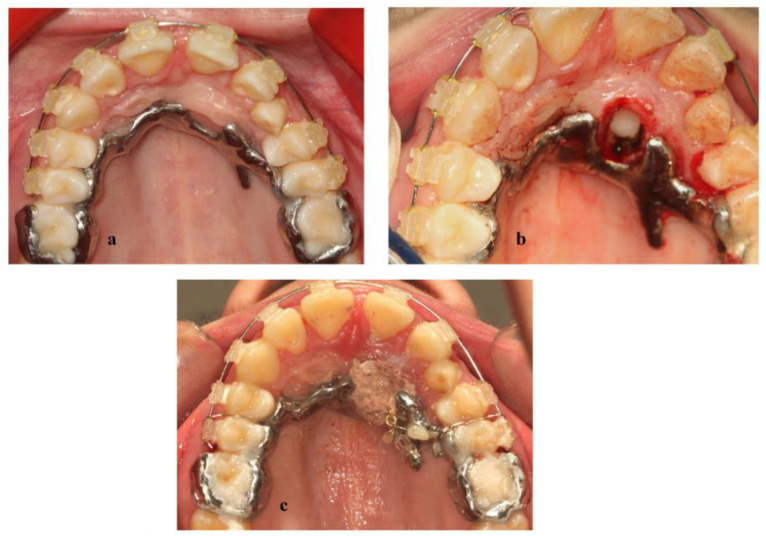
(**a**): The device fitted on the patient’s mouth, (**b**): surgical exposure of the canine, (**c**): gold chain bonded on the canine’s crown and surgical pack protecting the surgical site (patient 1).

Prior to the surgical procedure, the metal device was fitted and bonded on the patient’s upper arch with G-CEM Link-Ace, self-adhesive resin cement (GC Corporation, Tokyo, Japan). Under local anesthesia (3% solution of mepivacaine), soft tissue and bone, directly overlying the impacted canine, were removed and an orthodontic attachment was bonded on the canine’s crown. In order to protect the treated area, a surgical dressing was applied (Coe-Pak^®^, GC, Tokyo, Japan) for a week (Figure 5). 

**Figure 6:**
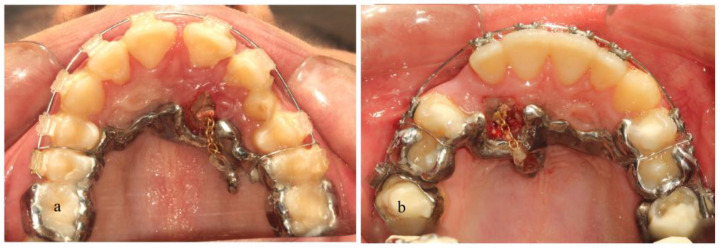
The gold chain apparatus mounted to the projection of the device, through an elastic chain ((**a**): patient 1, (**b**): patient 2).

**Figure 7:**
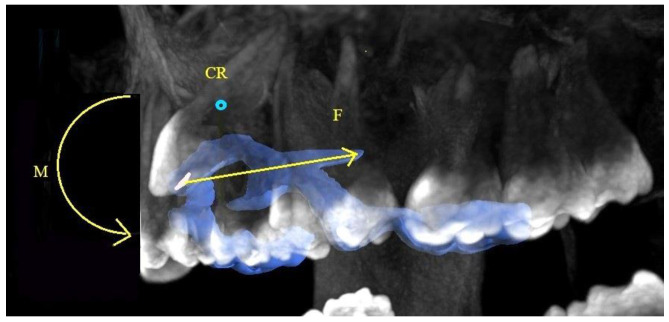
Force (F) delivered to the canine through the device and the corresponding moment (M), since the point of force application on the crown is lower and at distance from the center of resistance (CR) of the canine.

After the removal of the surgical pack, the chain apparatus was connected to an elastic chain mounted on the projection of the metal device and traction to the canine was started, delivering the desired forces and moments (Figure 6 and Figure 7).

## 3. Results

**Figure 8:**
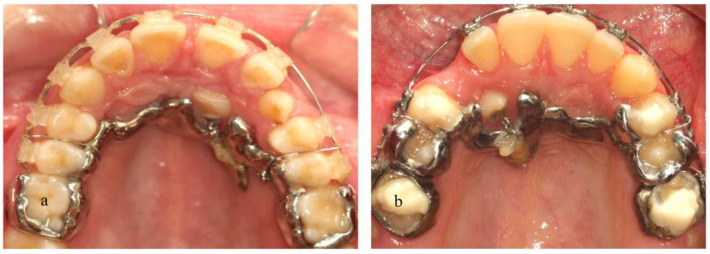
After 3 months, the canine’s crown appeared in the palate ((**a**): patient 1, (**b**): patient 2).

Within 3 months, after delivering traction to the canines, the crown appeared in the patients’ palate, in the predicted location (Figure 8). 

**Figure 9:**
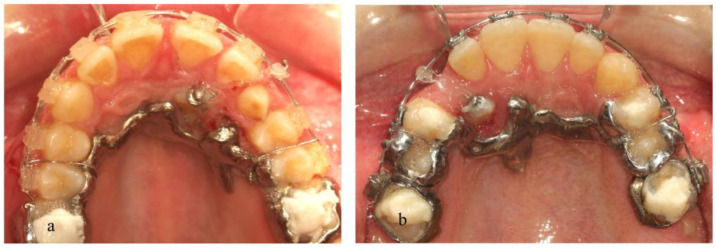
Traction of the canine to a labial direction ((**a**): patient 1, (**b**): patient 2).

After removal of the chain apparatus, an orthodontic button was attached to the labial surface of the crown and traction was delivered through an elastic chain to a labial direction (Figure 9). 

**Figure 10:**
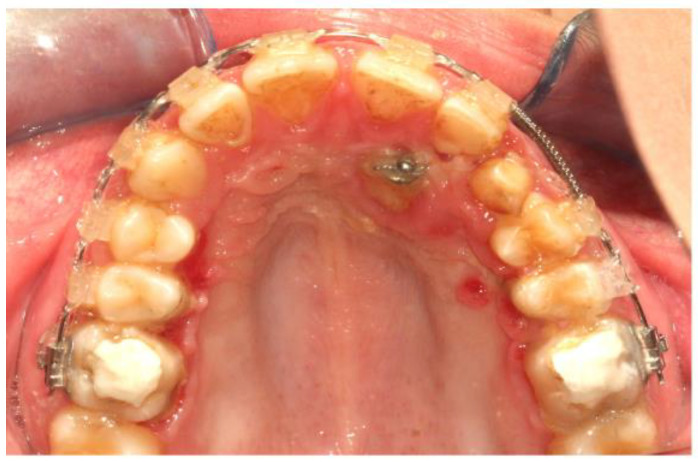
Right after the removal of the metal device. Notice the mild irritation of the palatal. mucosa (patient 1).

**Figure 11:**
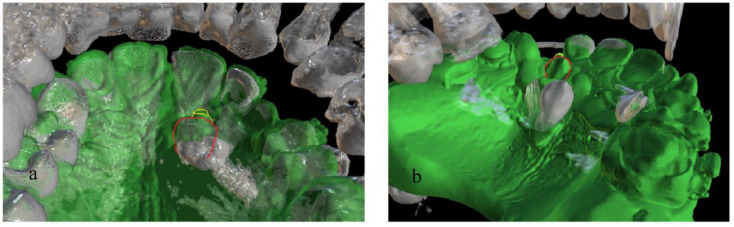
Superimposition of initial Cone-Beam Computed Tomography (CBCT) and stereolithography (.STL) file, after 4 months (3 months of traction with the device and 1 month of traction to a labial direction). Green color: .STL file from intraoral scanning after 4 months, red line: outline of the canine’s crown in the new position, yellow line: the bonded attachment. The canine is directed to its final position in the arch ((**a**): patient 1, (**b**): patient 2).

At that point, the metal device was removed from the patients’ mouth (Figure 10). The traction with a proper palatal direction, by the use of the device in the beginning, created the conditions for the safe final traction of the canines to the right position in the arch (Figure 11).

## 4. Discussion

The most common causes for canine impaction, as stated by Bishara [30], are localized pathologic conditions (arch length deficiency, prolonged retention of deciduous teeth, an alveolar cleft or cystic formation). It was also found by Jacoby [35], that in 85% of palatally impacted canines there is adequate space in the arch for eruption and the situation is attributed to some extra space in the maxilla created by either excessive bone growth, missing lateral incisors or premature eruption of the first premolar or the lateral incisor. In the first case presented, a prolonged retention of the deciduous canine was recorded, while in the second one the patient presented a skeletally-deficient upper jaw in the transverse plane. 

In the literature, there is concern that CBCT might not be significant for the opened or closed technique. In the pre-operative evaluations, no significant difference was found by Alqerban et al. [19], between the 2D and 3D information, regarding the type of treatment chosen (e.g., surgical exposure with or without attachment and canine extraction), or regarding the eruption technique (e.g., open vs. closed eruption). This was determined by the surgeon’s personal preference and experience regarding the best surgical approach. In agreement, Wriedt et al. [18] stated that proposed treatments for impacted canines do not differ, whether based on 2D or 3D images.

However, when it comes to spotting the exact point on the canine’s surface to place the eyelet of the chain apparatus and decide about the direction of traction, the CBCT is preferable to a panoramic radiograph [14,15]. CBCT images can increase the clinician’s confidence regarding the canine location, contact with the adjacent teeth and the presence or not of root resorption, thus leading to a successful operation [18,19]. Based on this assumption, in the presented cases, a CBCT imaging for locating the impacted canines was acquired, as it is indicated on the basis of research evidence [34]. This was combined with the .STL files, acquired by an intraoral scanning. This procedure poses certain advantages in the diagnosis and evaluation of treatment outcomes in orthodontic therapy [36,37]. On the digital model that was created, with the help of software (Blue Sky Plan^®^, Version 4.9.4, Blue Sky Bio, LLC, Libertyville, IL 60048, Illinois, USA), a device for surgical exposure and traction of the canine was designed.

Special cantilever springs have been proposed as a multi-tool in orthodontics [38] and are used for the treatment of palatally impacted canines, either inserted to the buccal auxiliary tubes of the molar band [39] or to the lingual sheath [40] and attached to the canine. However, they usually have a complicated design, which poses certain difficulties in construction, while multiple loops can be greatly difficult to adjust in clinical practice. This is left to the technician’s or doctor’s experience. Activation of such devices is often attempted inside the patients’ mouth and this could be annoying or potentially dangerous. Reducing the wire’s diameter in order to control the force applied [32] makes the appliance more prone to breakage. The position of the springs in the mouth can traumatize the tongue or cheeks. Furthermore, possible detachment of the eyelet of the traction apparatus can lead to an emergency situation. Of course, it should be underlined that even difficult cases of impacted canines can be tackled with conventional mechanics, such as the double-arch technique [41]. In this technique, a properly designed stainless steel auxiliary arch is utilized in conjunction with a basic stainless steel arch wire, in order to deliver proper traction to an impacted canine.

On the other hand, a digitally designed and 3D-printed appliance or surgical guide, manufactured by combining CBCT, intraoral scanning, CAD and 3D printing technology, has certain advantages: improvement of accuracy, reduced operating time and errors and more predictable treatment results [42]. Even the duration of the surgical procedure is reported to be reduced and less invasive [43].

In this way, a homogenous, minimal and smooth construction, with a precise fit on the teeth and mucosa surfaces is acquired, like the one used in these cases. This guarantees easy cleaning and also a pleasant feeling to the patient. In fact, the particular device with the semi-circular construction indicates the exact point on the palatal mucosa for the surgeon to elevate the flap. With the help of the underlying CBCT, when the mucosa and bone are removed, the exact point on the canine’s crown where the eyelet of the chain apparatus will be bonded is revealed. Furthermore, a projection is incorporated in the device which is used to mount the elastic chain for the traction of the canine. The shape, size and thickness of the device, and especially of the semicircular and projection part, is designed after analyzing the proper direction of the traction, to ensure the best results of guiding the canine in the proper place, in the initial phase of the treatment.

Regarding the material used, 3D-printed Co–Cr alloy with Selective Laser Melting (SLM) technology (direct printing of a metal component, fusing metal powder in layers, by a high-power laser beam), displayed greater electrochemical stability, high polarization resistance, corrosion potential, and pitting potential when compared to conventional techniques [44,45]. 

Irritation of the soft tissues (Figure 10) is a potential adverse effect of the proposed device, as it is in contact with the palatal mucosa. Thorough cleaning of the device using interdental brushes of proper size, mouthwash solutions and perhaps other adjuvant treatment such as photodynamic therapy (PDT) [46], is of great importance. The detailed and frequent inspection of the device and the proper activation is also essential. Future research should focus on digitally defining the pathway of orthodontic traction of an impacted canine and on implementation through 3D-printed devices. 

## 5. Conclusions

Successful exposure and traction of palatally impacted canines can be aided by devices which are computer designed and manufactured, using 3D printing technology. Digital procedures in diagnosis like obtaining a CBCT and an intraoral scanning, substantially accommodate this effort.

## Data Availability

Data available upon reasonable request.
